# Re-Mind the Gap! Insertion – Deletion Data Reveal Neglected Phylogenetic Potential of the Nuclear Ribosomal Internal Transcribed Spacer (ITS) of Fungi

**DOI:** 10.1371/journal.pone.0049794

**Published:** 2012-11-19

**Authors:** László G. Nagy, Sándor Kocsubé, Zoltán Csanádi, Gábor M. Kovács, Tamás Petkovits, Csaba Vágvölgyi, Tamás Papp

**Affiliations:** 1 University of Szeged, Faculty of Science and Informatics, Department of Microbiology, Szeged, Hungary; 2 Clark University, Biology Department, Worcester, Massachussetts, Unikted States of America; 3 Institute of Biology, Department of Plant Anatomy, Eötvös Loránd University, Budapest, Hungary; 4 Plant Protection Institute, Centre for Agricultural Research, Hungarian Academy of Sciences, Budapest, Hungary; University of California Riverside, United States of America

## Abstract

Rapidly evolving, indel-rich phylogenetic markers play a pivotal role in our understanding of the relationships at multiple levels of the tree of life. There is extensive evidence that indels provide conserved phylogenetic signal, however, the range of phylogenetic depths for which gaps retain tree signal has not been investigated in detail. Here we address this question using the fungal internal transcribed spacer (ITS), which is central in many phylogenetic studies, molecular ecology, detection and identification of pathogenic and non-pathogenic species. ITS is repeatedly criticized for indel-induced alignment problems and the lack of phylogenetic resolution above species level, although these have not been critically investigated. In this study, we examined whether the inclusion of gap characters in the analyses shifts the phylogenetic utility of ITS alignments towards earlier divergences. By re-analyzing 115 published fungal ITS alignments, we found that indels are slightly more conserved than nucleotide substitutions, and when included in phylogenetic analyses, improved the resolution and branch support of phylogenies across an array of taxonomic ranges and extended the resolving power of ITS towards earlier nodes of phylogenetic trees. Our results reconcile previous contradicting evidence for the effects of data exclusion: in the case of more sophisticated indel placement, the exclusion of indel-rich regions from the analyses results in a loss of tree resolution, whereas in the case of simpler alignment methods, the exclusion of gapped sites improves it. Although the empirical datasets do not provide to measure alignment accuracy objectively, our results for the ITS region are consistent with previous simulations studies alignment algorithms. We suggest that sophisticated alignment algorithms and the inclusion of indels make the ITS region and potentially other rapidly evolving indel-rich loci valuable sources of phylogenetic information, which can be exploited at multiple taxonomic levels.

## Introduction

Phylogenetic markers with length mutations (insertions and deletions, i.e. indels), introns, ribosomal RNA regions or non-coding sequences, are popular targets for many phylogenetic studies, because of appropriate variability and/or easy accessibility even from old herbarium specimens, yet the challenges in alignment and phylogenetic inference imposed by indels are not completely resolved. The evolution of indels and nucleotide substitutions share largely the same patterns, but the mechanisms generating indels are more diverse, including transcriptional slippage, the formation of hairpins, deletions, insertions, inversions and duplications [1]. According to the general view, indels – especially longer ones, which are incompatible with constraints on secondary structure – are less frequent than nucleotide or amino acid replacements [2], [3], although this view has been challenged [4]. If true, however, gaps of unambiguously aligned regions would contribute a valuable source of phylogenetic signal for higher-level evolutionary studies. Despite the preponderance of indels in ITS alignments, these have hardly been recognized as a potential source of phylogenetic information in fungi (for exceptions see [5], [6], [7], [8]), unlike in introns, where the value of length-mutations is more widely recognized in plants and animals [3], [9], [10], [11], [12], [13]. Indels in intron sequences have often been reported to be less [14], [15] or even not homoplasic [3] and have lower rates of evolution [2], [3]. Although many gaps are phylogenetically informative, their usage in phylogeny reconstruction raises several problems, including differences in indel birth rate and indel length between different regions of DNA and the ability of phylogenetic methods to capture these. Independence of characters may also be violated if multi-residue indels are treated as multiple evolutionary events, such as in fifth character-state coding in parsimony analyses (for a solution see below), or when independent insertion/deletion events cannot be recognized due to a shared 5′ and 3′ end. Building on these observations, a higher success of phylogenetic reconstruction was noted in several studies [3], [15], [16], [17], [18], however, none of these studies addressed the contribution of gap data across a range of evolutionary distances or alignment algorithms.

It is widely accepted that the proportion of correctly inferred positional homologies is the most important determinant of the outcome of phylogenetic reconstruction [19], [20], [21], [22], [23], [24]. Improving indel placement accuracy has been one of the major lines of experimentation in the development of alignment algorithms, with solutions ranging from penalizing indels [25] to modelling their evolution by Markovian models allowing single or multiple insertions/deletions at a time [22], [23]. Information on secondary structure, and thus constraints imposed on where indels are tolerated in a sequence, has also been incorporated [26], [27]. How these affect indel placement in real datasets, and when should indels be included, excluded or treated as missing data in phylogenetic analyses has not yet been fully explored.

A number of approaches have been proposed to handle indel-rich regions of alignments [18], [28], [29], [30], [31], [32], ranging from the complete exclusion of gapped sites from the phylogenetic analysis, to treating them as missing data or recoding as presence/absence characters and including them in the analyses. Most approaches for cleaning alignments from ambiguously aligned regions use gappiness as a proxy for potentially mis-aligned and/or saturated blocks of alignments [28], [32]. Of the available approaches, the simplest is to treat indels as a fifth or 21th character state for nucleotides and amino acids, respectively. However, this approach treats multi-residue indels as multiple evolutionary events, whereas it is probably more biologically realistic to handle such indels as one event only. Another approach, INAASE, produces a separate matrix of recoded gap characters without violating positional homologies [29]. Both approaches can only be used in parsimony. The output of simple indel coding and complex indel coding [31] can, however, be analyzed by model-based phylogenetic methods including Bayesian inference, since these convert the array of independent indel events in the alignment to a binary (presence/absence) or multistate matrix. In simulation studies, the accuracy of simple and complex indel coding were almost identical, both outperforming other approaches, including the exclusion of gapped sites [18]. As a surrogate to the traditional 2-phase approach of phylogeny reconstruction and optionally indel coding, simultaneous alignment and phylogeny inference with elaborate insertion-deletion models is becoming accurate and computationally tractable [20], [21], [23]. Despite their great theoretical advancement, however, computational tractability have so far prevented these to become routine approaches in phylogeny reconstruction of tens or hundreds of sequences (for an example, see [5]).

Internal transcribed spacers (ITS) of the nuclear ribosomal RNA cluster are the most frequently sequenced phylogenetic markers in fungi [33], and a considerable sequencing effort is put into ITS also in plants [34], [35], [36], [37], [38], and to a minor extent in bacteria [39] and animals [12]. Among the major sources of the popularity of the ITS region are the high copy number and consequently, the easy accessibility via PCR even from fragmentary or badly preserved samples, widely tested primers, which amplify the ITS across all major fungal groups [40]; a general length of 400–800 bp, which makes it easy to sequence as well as proper phylogenetic resolution at the species level. These factors have contributed to ITS becoming the most widely used locus in species-level phylogenetic, metagenomic [41] and environmental sequencing projects in mycology and botany [42], [43], [44], [45], [46], [47]. Two recent surveys found that the ITS is sequenced in over two thirds of plant and fungal phylogenetic studies [34], [36]. Furthermore, ITS is the most popular marker for identifying pathogenic (e.g. [48]) and other fungi [49] and became the official barcode marker for fungi. Bioinformatic tools [50], [51], [52] developed for the reliable taxonomic affiliation of environmental and insufficiently identified sequences are also based mostly on the ITS or its fragments, mostly the ITS2, and include a chimera checker [53], pipelined processing and identification of ITS sequences [43], [44], [54], a workbench for annotation and identification [55] and even a biweekly service to monitor insufficiently identified ITS sequences in international sequence databases [56].

However, two major lines of criticism exist against the ITS locus of the nrDNA repeat. One is the difficulty of multiple alignment of ITS sequences of distantly related species [34], [36], [57], [58]. The whole ITS locus is composed of two non-coding regions, the ITS1 and the ITS2, which flank the fragment encoding the 5.8S ribosomal RNA subunit. Of these, the 5.8S region is highly conserved even across kingdoms, whereas the ITS1 and ITS2 regions are highly variable, rapidly accumulating nucleotide substitutions and length-mutation events. The variability of the ITS1 and ITS2 regions in animals is even more extreme, limiting its use even at the species level. Indels make multiple alignment of divergent ITS sequences challenging, due to a high risk of inferring false positive positional homologies and increasing artifactual support for incorrect relationships [73]. The second criticism against ITS is the loss of phylogenetic resolution in intergeneric comparisons and above [47], due to high substitution rates resulting in high homoplasy, which mask the phylogenetic signal for early evolutionary events. This has led to a general reluctance to use ITS at all above the species level, or the exclusion of the majority of the variable regions in ITS1 and 2 [5], [36], [59], [60], and a general view that ITS is hardly suitable for resolving relationships at higher levels.

In this study we set out to examine the impact of various indel handling approaches to the outcome of phylogenetic inference from fungal nrDNA ITS sequences. We test whether and how the incorporation of the phylogenetic information of gaps alters the resolution and branch support in Bayesian phylogenetic analyses. More specifically, based on previous studies of intron sequences, we hypothesize that the use of indel data extends the phylogenetic utility of ITS to include evolutionary divergences above the level of interspecific comparisons. Further, we investigate whether the inverse approach, the exclusion of “ambiguously aligned regions” in turn improves phylogenetic inference. All the before mentioned questions are explored using published fungal ITS datasets, across four alignment algorithms with different theoretical background and implicit assumptions.

## Methods

### Datasets, Alignments and Coding of Indels

We collected published ITS alignments of fungi in the wide sense (including Oomycota) from major mycological journals and assembled the published ITS dataset by downloading the sequences from GenBank, the whole alignment from TreeBase, or requested the dataset from the authors of the studies. Datasets were checked for sequence quality and length, those containing poor quality or overly short sequences (<50% of the average length) were excluded. We also omitted sequences which have been uploaded to GenBank with the “ambiguously aligned” regions deleted, which would be reconstructed as biologically nonsense gaps in the alignments. Datasets were chosen to represent a wide range of fungal groups, covering all phyla and comprise several taxonomic ranges, from species- to phylum level. Two new datasets were also assembled (published as part of this study, TreeBase Accession No: 12470). In one case, the Agaricales ITS dataset of Matheny et al [61], we pruned several taxa from the alignment to maintain computational tractability. All datasets were aligned by four alignment programs, ClustalW 1.83 [59], MAFFT-X-INS-i 6.717b [25], Probalign 1.3 [26] and PRANK_+F_ 1.058 [62]. The four alignments represent four ‘milestones’ in multiple sequence alignment development, and simulation studies show higher accuracy for each one over its predecessors [16], [19], [63], [64], [65], [66]. Of these, the latest is PRANK, which has been shown to outperform other methods with regard to accuracy in indel-rich sequences [19], [63]. As fine tuning of alignment parameters was out of the scope of our study, all programs were launched with the default parameters. MAFFT was launched with MXSCARNA [62] to estimate secondary structure, based on the Four-Way Consistency objective function [27]. In PRANK_+F_ we invoked the_+F_ option to fix already inferred indels at their place and avoid another indel to be inferred in an overlapping position during the second recursion of the algorithm [26]. This reflects the notion that indels introduced along one branch of the phylogeny are from an indel event another branch, even if they overlap, and thus produces more gappy, but potentially more accurate alignments. Overhanging sequence fragments of both termini were trimmed from all alignments, the remaining sequences whenever possible, to start with the CATTA motif at the end of the 18S rRNA region and end with the first nucleotide of the 28S rRNA region.

We coded all indels in each alignment by means of the simple indel coding algorithm as implemented in FastGap [62], which converts all indels with different starting and/or end positions to a matrix of binary presence/absence characters. Indels falling within the range of a longer indel were coded as unknown character states. Leading and trailing gaps of the alignments were scored as missing data. Although simple indel coding is not the most advanced approach for indel coding, it has been shown to approach the performance of modified complex indel coding very closely [67].

Ambiguously aligned regions were identified by using GBlocks 0.91 [18], [28] with a “less stringent” set of parameters: the maximum number of contiguous non-conserved positions was set to 15, the minimum length of a block is five characters, and allowing half of the sequences to contain gaps at each site. GBlocks eliminates poorly aligned and/or saturated positions flanked by conserved regions. Its parameters define what percent of gaps and non-contiguous positions are allowed in the retained blocks. In this study we chose parameters that allow some of the gapped sites to be retained, thereby obtaining a conservative estimate of the effects of excluding ambiguously aligned regions from the alignments.

### Phylogenetic Analyses

To examine the impact of various gap treatments, we compared the resolution and nodal support of trees inferred when treating indels as missing data, recoding them as presence/absence characters or deleting gapped sites from the alignments. Thus, for each alignment (four for each dataset), we prepared three nexus input files for phylogenetic analyses, one from nucleotide data only, one from alignments with the ambiguously aligned sites excluded and the third from nucleotides combined with recoded indels (for coding, see above). For the best scoring alignment algorithm, we performed a fourth set of analyses, in which trees were inferred from the indel matrix only.

We used the parallel version of MrBayes 3.1.2 [32] for estimating trees and support values for each dataset, under various alignment algorithms and gap treatments. Nucleotide-only analyses were not partitioned, combined nucleotide plus indel analyses were divided into two partitions. Within the nucleotide alignments, gaps were treated as missing data (i.e. not as a fifth character state). We applied the GTR+G model and a two-parameter Markov model (Mk2 Lewis) for nucleotide data and indel matrices, respectively. Although over-parameterisation in some cases is probable with these settings, we decided to routinely apply these models for consistency among datasets. For the indel matrices, a correction for constant characters not included in the matrix (as implemented in MrBayes) has been applied during the analyses. MrBayes was launched with default priors, two replicated runs with one cold and three incrementally heated Markov chains per replicate. The Markov chains were run for five million generations uniformly, with sampling each 100^th^ generation. We used topological convergence as judged from the average standard deviation of split frequencies as a conservative measure of convergence of the MCMC analyses. Topological convergence was judged sufficient when the average standard deviation of split frequencies dropped below 0.01. The burn-in was set to three million generations, or established by inspecting convergence. Trees remaining after the exclusion of the burn-in phase were used to compute 50% majority rule consensus trees and Bayesian posterior probabilities (hereafter PP).

### Comparison of the Phylogenetic Signal Under Various Indel Treatments

We took two different approaches to characterize the effects of various treatments of indels in the phylogenetic analyses. First, as a crude measure of the amount and strength of phylogenetic signal, we calculated the resolution and the mean of the posterior probabilities of resolved nodes for each consensus tree. We observed that tree resolution and the mean of the PP-s are in a reciprocal relationship when there are weakly supported nodes in the phylogeny. This is because weakly supported nodes either collapse to polytomy or receive low PP values when get resolved. A polytomy does not contribute anything to the mean of the PP-s, but reduces resolution, whereas a resolved node with a low PP likely reduces the mean of PP-s but increases resolution. We overcame this problem by taking the product of the two measures. Resolution was calculated as *m/(n-2)* where *m* is the number of resolved nodes and *n* is the number of tips of the tree.

To examine how different alignment algorithms and the inclusion of indels affect the phylogenetic signal at different taxonomic ranges, we plotted PP-s as a function of node depth (distance from the tips). We divided the range of node depths to 800 discrete categories and calculated the mean of the PP-s falling in each of the categories. Node depths were computed for each node as the mean of the path lengths leading from the node of interest to all of its descendant leaves. If ITS sequences lose informativeness above the species level, we expect the PP-s to decrease sharply toward higher node depth values. Further, if indels are more conserved than nucleotides, the inclusion of indels in the analyses is expected to add increasingly more to the posterior probabilities at higher evolutionary divergences. These analyses have been performed both for a collection of all resolved nodes and for those that are congruent (i.e. have the same set of descendants) on the pair of consensus trees inferred from nucleotide data and nucleotide plus indel data. A set of perl scripts written to perform the abovementioned analyses are available from the authors upon request.

## Results

One hundred and fifteen datasets, spanning several taxonomic ranges (species to phylum level) and fungal groups (Ascomycota, Basidiomycota, Glomeromycota, Mucoromycotina from the group of true Fungi and Oomycota) were collected and analyzed in this study. The classification, the approximate taxonomic range, the number of taxa and literature references are presented in Table S1. We computed four alignments for each dataset and ran three analyses for each alignment (i) one using the nucleotide data only (ii) one with the nucleotide and indel matrix combined and (iii) one where the ambiguously aligned regions were deleted. In addition, we ran a 4^th^ set of analyses in which only gap data, recoded from PRANK_+F_ alignments, were used. Some of the analyses failed to complete or converge significantly (i.e. average standard deviation of split frequencies not dropping below 0.01), these have been omitted from later comparisons.

**Figure 1:**
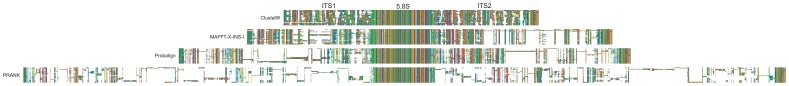
An example of differences in indel placement and alignment overmatching under various alignment algorithms in a family/genus level dataset. Three major features, the internal transcribed spacer 1, the fragment coding for the 5.8S ribosomal RNA and the internal transcribed spacer 2 are discernable, of which the 5.8S fragment is highly conserved, whereas ITS1 and ITS2 are rich both in nucleotide substitutions and length-mutations. Data are from Lutzoni et al [18].

Of the four alignment algorithms, ClustalW produced the shortest (481–1648; mean: 726.41), while PRANK_+F_ produced the longest alignments (508–5701; mean: 1422.51) on average. This difference was caused by both the number of recovered indel events and the length of individual indels (Table 1, Fig. 1). The most extreme alignment lengths were inferred for two, taxonomically divergent datasets spanning the Basidiomycota and Agaricales (5701 and 4209 sites, respectively [68], [69]) under PRANK. Differences in alignment length increased significantly with the increasing evolutionary divergence covered.

**Table 1:** Number of nucleotide and indel characters and parsimony informative sites under the four alignment methods examined in this study. Minimum and maximum values are given in parentheses.

	Nucleotide alignment				Indel matrix	
	Mean length	No. of variablesites	No. of parsimony informativesites	No. of variable/No. of parsimonyinformative sites	Mean No. ofindel characters	No. of variable/No. of parsimonyinformative sites
ClustalW	726.41 (481–1648)	399.67 (69–1006)	320.38 (36–685)	0.785	216.19 (6–780)	0.614
MAFFT-X-INS-I	846.57 (494–1934)	385.68 (71–907)	281.47 (38–637)	0.726	218.22 (6–711)	0.565
Probalign	948.35 (471–2416)	395.35 (76–1205)	272.71 (39–719)	0.702	268.9 (11–876)	0.551
PRANK_+F_	1422.51 (508–5701)	397.58 (70–1310)	257.19 (36–674)	0.674	345.4 (9–1449)	0.534

Indel lengths showed a strongly left-skewed distribution with long right tails, which was rather uniform across alignment algorithms (Fig. S1). Single nucleotide indels (both parsimony informative and uninformative) were far the most abundant of all length categories. Indels longer than 20 bases were rare, although their absolute number was highest in PRANK_+F_ (on average 18.51 and 13.79 bases for parsimony informative and uninformative indels, respectively, ANOVA p-value<0.001). Mean length of parsimony informative indels were rather uniform among ClustalW, MAFFT-X-INS-I and Probalign (4.75, 6.99 and 6.77), whereas the mean length of parsimony uninformative indels in MAFFT-X-INS-I was outstanding: 11.76 versus 5.53 and 8.02 for ClustalW and Probalign. Interestingly, the number of parsimony-informative singleton gaps was greater in ClustalW and MAFFT-X-INS-I alignments than that of parsimony-uninformative indels, whereas Probalign and PRANK_+F_ alignments showed an opposite pattern. On average, parsimony informative indels inferred by ClustalW, MAFFT-X-INS-I and Probalign were longer than parsimony uninformative ones (Table 2).

**Table 2:** Mean indel lengths under the four alignment methods examined in this study.

	Parsimony informative	Parsimony uninformative
ClustalW	4.75	5.53
MAFFT-X-INS-I	6.99	11.76
Probalign	6.77	8.02
PRANK_+F_	18.51	13.79

**Figure 2:**
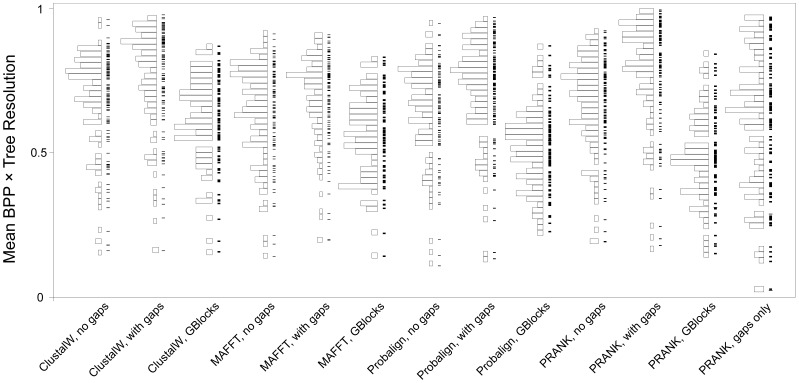
The impact of various gap treatments on ‘tree support’ calculated as the product of 50% majority rule consensus tree resolution and the mean of the posterior probabilities of resolved nodes. Thus, for instance, a value of 1.0 means that the consensus tree is fully resolved and the posterior probability is 1.00 for each node. Each panel reflects the distribution of ‘tree support’ values for each of the analyzed datasets (115 on average, minus failed analyses). The figure shows that the inclusion of indel data increases tree support under all alignment algorithms, except for MAFFT-X-INS-I, whereas the exclusion of ambiguously aligned sites (by GBlocks) reduces tree support for all alignments. The differences are most pronounced for PRANK_+F_, which inferred on average the highest number of indels and gapped sites (see text).

The impact of indel characters and the exclusion of ambiguously aligned sites on phylogeny reconstruction was measured by the difference in tree resolution and posterior probabilities they cause. To this end, we calculated the product of tree resolution and mean posterior probabilities for each dataset as a proxy for ‘tree support’. The inclusion of indels in the phylogenetic analyses shifted the distribution of tree support values considerably to higher values for ClustalW, Probalign and PRANK_+F_ with the highest difference observed for PRANK_+F_ alignments, whereas the difference was negligible for MAFFT-X-INS-I alignments (Fig. 2). On the other hand, the exclusion of ambiguously aligned regions uniformly decreased the resolvability and posterior probabilities of nodes. When only indel matrices (from PRANK_+F_) were analyzed, the distribution of tree support showed an even distribution over the range [0,1]. We found that very low values correspond to indel-poor alignments, providing only a few characters to analyze.

**Figure 3:**
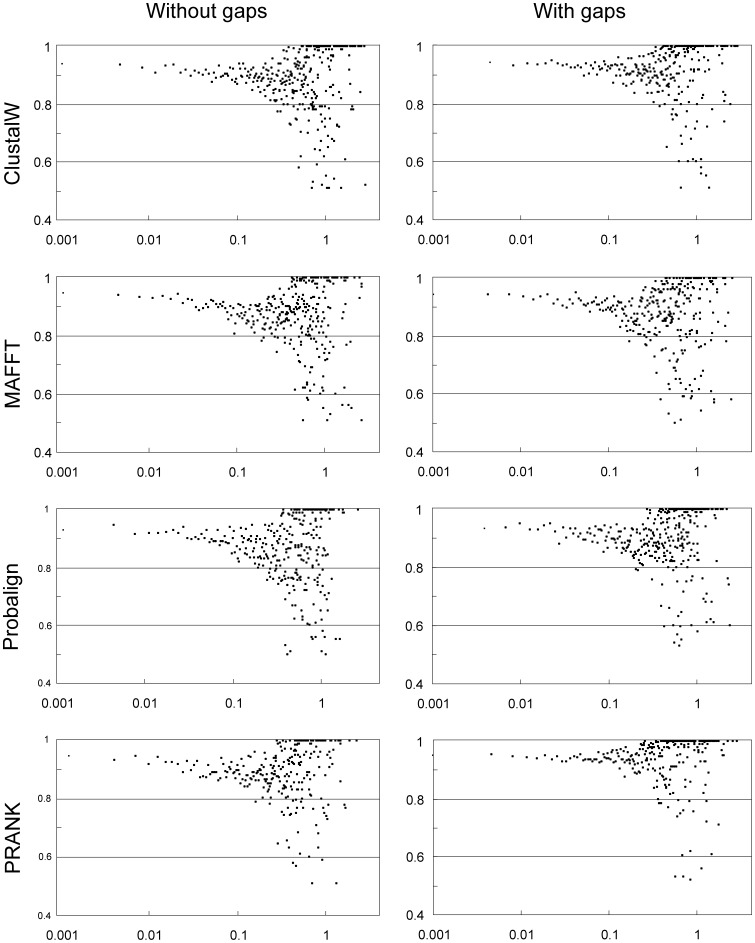
The phylogenetic utility of the ITS region as a function of evolutionary divergence. To test whether indel data extends the resolution power of the ITS locus above the species level (i.e. above intrageneric comparisons), we depicted node resolvability as a function of node depth, calculated as the mean of the path lengths between the node of interest and its descendant leaves on each consensus tree. The range of node-depth values was divided to 800 discrete categories (200 and 400 categories produced the same distributions). Depicted are the means of the Bayesian posterior probabilities of the nodes falling in a specific depth-category (drawn on log scale). Lower values towards higher node-depths signify the decay of phylogenetic signal in the alignments at earlier evolution events. However, it is evident that the addition of indel data decelerates the rate of this decay, except for MAFFT-X-INS-I, for which the effect is hardly noticeable. We found that the high number of values at 1.0 point to depth-categories with a single value, contributed by nodes strongly supported under all settings (very distinct lineages, such as outgroups). For comparison, we made a rough estimate between the relationship of taxonomic range and node depths. Node depth values 0.04–0.23 correspond the genera (although the most recent common ancestor of *Russula* was on average 0.41 from the tips), 0.22–0.55 to families, whereas 0.5–1.33 to orders. The mean path length to the ancestor of the Basidiomycota was (James et al 2006) 2.3 unit.

The relationship between node depth – and thus taxonomic range – and the contribution of indel data was examined by extracting the mean of node-tip distances and the corresponding posterior probability values for each resolved node. These data were collected for each dataset under each alignment algorithm, yielding a total of 4749/5195, 4308/4646, 3833/5208 and 4451/5010 nodes for the “nucleotide only”/”nucleotide plus indel” analyses of ClustalW, MAFFT-X-INS-I, Probalign and PRANK_+F_ alignments, respectively. Their distribution as a function of node depth is depicted on Fig. 3. The inclusion of indels shifted the distribution of posterior probabilities upwards, except in MAFFT-X-INS-I alignments, where this effect was hardly observable. The difference is most pronounced in PRANK_+F_ and Probalign alignments, where a regression analysis assuming linear decay of tree support as a function of evolutionary divergence suggests that the tempo of the breakdown of phylogenetic signal with increasing node depth is considerably slower when gaps are included in the analyses (Table S2).

The number of nodes congruent between the trees obtained with and without indels was 3102, 2921, 2379 and 3056 for ClustalW, MAFFT-X-INS-I, Probalign and PRANK_+F_, respectively (Fig. S2). Again, the results imply a stronger phylogenetic signal of nucleotide plus indel analyses over their nucleotide-only counterparts. Differential contribution of indel data at different node depths could not be unambiguously traced, probably due to the rapid decrease in the number of nodes towards more ancient divergences (which is consistent with both the Yule and BD models of speciation). Nevertheless, for the overwhelming majority of congruent nodes, posterior probabilities on indel-including trees were higher than PP-s on trees inferred without the indel data (Fig. S2).

## Discussion

The utilization of alignment gaps is promising for many phylogenetic markers, not only because it represents an additional source of information from the same sequenced loci, but also since length mutations may be more conserved than nucleotide changes [1], [2], [59]. In this study we examined how the resolution and branch support values of Bayesian consensus trees change under different gap treatment strategies found in the literature, including simple indel coding [3], GBlocks curation of alignments [31] and treating them as missing data. Our results suggest that the inclusion of indels enhance the resolution power of the alignments and that combined nucleotide plus indel analyses of ITS sequences provide support at a greater spectrum of evolutionary depths as compared to the routine approach of using nucleotides only. Indel data increased branch support and consensus tree resolution for practically all genus, family, order and even phylum level data sets in our analyses. As expected, the mean of posterior probabilities showed a negative correlation with node depth, that is, the greater the evolutionary distance, the lower are the posterior probabilities due to the gradual decay of the phylogenetic signal in the sequences. However, this breakdown is significantly slower when gaps are included in tree estimation. This contradicts the prevailing view in mycology, which regards the ITS locus as a marker of species-level phylogenetics (e.g. [28], and suggests that gaps in ITS alignments represent an additional source of phylogenetic information, that is more conserved than nucleotide changes. The lower number of gap characters as compared to variable nucleic acid sites implies that the rate at which length-mutations (i.e. indels) accumulate in the ITS1 and ITS2 loci, is lower than that of nucleotide substitutions, which is consistent with patterns found for introns of protein coding genes [2], [3], [36]. However, while the four alignment methods inferred nearly identical numbers of variable nucleic acid sites, the mean number of recovered indel events per alignment differed significantly; ranging from 216.19 for ClustalW and 345.4 for PRANK_+F_. This, although concordant with the general view that indels are more conserved than nucleotide substitutions, implies considerably different conclusions about the extent of conservation of the two types of characters and highlights the potential impact of alignment assumptions on the conclusions about conservation. Nevertheless, indel data represent a valuable source of phylogenetic information that is (slightly) more conserved than nucleotide substitutions in the ITS region. With the emerging interest in finding new sources of conserved phylogenetic information sparked by recent large scale phylogenetic [11], phylogenomic [69] as well as several order- and family level studies, indels of ITS or other rapidly evolving genes [70], [71] represent good candidates to become involved in future higher level phylogenetic studies. Another important factor limiting the success of reconstruction of early relationships from indel-rich markers is taxon sampling density. Simmons and Freudenstein [72] noted that the phylogenetic signal may be masked significantly when the proportion of nodes recovered along a phylogenetic path is low. That is, increased taxon sampling provides the intermediate states of both nucleic acids and indels at intervening nodes, thus facilitating alignments and phylogeny reconstruction.

A common strategy to by-pass alignment difficulties is to exclude “ambiguously aligned” regions from the analyses, either manually or using a computer program [18], [28]. This has been suggested to improve phylogenetic signal by eliminating much of the alignment noise resulting from overly variable regions of distant sequences [28], [32], [73], although contradicting evidence has also been published [32], [64]. In this study, we examined how the phylogenetic signal in ITS alignments change when gapped sites are excluded using a conservative approach. The deletion of gapped sites universally decreased tree resolution and branch support and the decrease in tree support was bigger for more sophisticated alignment algorithms (lowest in ClustalW, highest in PRANK_+F_). We believe that the explanation for this is that, since computerized methods use gap content per alignment column as a proxy for being ambiguously aligned, not the actual rate (e.g. substitutional saturation) of the given column, a greater portion of sites is excluded from more gapped (i.e. less condensed) alignments, which necessarily entails more properly aligned sites to be deleted also. Although our study was not designed to objectively benchmark alignment accuracy, simulation studies provide robust evidence for the accuracy of indel placement by various alignment algorithms [16], [18], [19], [62], [64], [65], [66], which correlate well with our observations made on the basis of tree resolution and branch support for empirical datasets. The increasing loss of resolution for more gapped alignments highlights a very important and – in our opinion, generally overlooked – aspect of data exclusion, namely that gap content alone may not be a good proxy for identifying alignment regions of low quality. Although the distribution and correlation of the rate of indel events and nucleotide substitutions certainly have a bearing on this question, such information is hardly available in the literature. Recently developed alignment methods with more biologically realistic gap placement may infer positional homologies even in highly gapped regions of the alignment, which, when eliminated from the analysis, results in a loss of phylogenetic resolution. In previous studies, the positive effects of excluding ambiguously aligned regions were obtained by using alignments produced by the least sophisticated algorithms tested here. Because those methods are known to infer a high number of false positive positional homologies [16], [19], [62], [63], [64], [65], [66], which can mimic substitutional saturation, it is possible that the beneficial effect of deleting such sites may come from the elimination of non-homologous sites, which might explain the alleged disagreement about the significance of data exclusion found in the literature [18], [28], [63], [64], [73]. Consistent with this notion, the exclusion of gapped sites resulted in a steady decrease in tree support when the alignments were known with certainty [32]. Therefore, it would be interesting to examine whether the same results can be obtained under alignment settings inferring a much fewer false positive positional homologies.

One evident limitation of our approach is that the trees inferred under various alignment methods and gap treatments cannot be validated against the “true tree”. However, pervasive conflict between nucleotide and indel data, which may in part be caused by abundant alignment noise, and could be detectable by poor convergence of MCMC analyses and/or expansive polytomies, has not been implicated by our results. Given the paucity of model-based phylogenetic software capable of handling binary data, formal testing of conflict between nucleotide and indel matrices is not possible. An intriguing future extension of this work would be to examine the extent and nature of conflict between different types of data from the same locus. Nevertheless, manual comparison of trees inferred from indel and nucleotide data for individual datasets could serve as a surrogate of formal conflict testing.

In this study we used simple indel coding [15] combined with Bayesian inference to evaluate the phylogenetic signal in indel data due to the size and amount of datasets at our disposal. As a surrogate to the two-phase approach we applied, simultaneous alignment and phylogeny inference approaches [20], [22], [23] provide a natural way to integrate indel information into phylogeny inference, without the need for separate handling and recoding of indels. Simultaneous sampling of trees and alignments is also less prone to the violation of positional homology, at the cost of increased computational demand.

The internal transcribed region represents the most ubiquitously used sequence fragment for phylogenetic analysis, sequence-based species identification, including environmental sequencing and barcoding in fungi. Recently, the widespread application of pyrosequencing for the characterization of fungal communities multiplied the interest in ITS, which is also reflected by the number of available software tools and databases (e.g. [18]). It has long been recognized that, like other rapidly evolving non-coding sequences, the ITS region accumulates phylogenetically informative indels [5], [7], [34], [36], [56], yet this type of information is almost completely neglected in all of the abovementioned aspects of current mycological praxis. In addition to *in silico* analyses requiring multiple alignments, indel information could be exploited in pairwise comparisons conducted in certain species identification procedures and barcoding [8], as well as in vitro detection of species by specifically designed primers. In this study, we found that alignment gaps can be used to augment the phylogenetic signal in fungal ITS sequences under a range of conditions, which is consistent with the results of both empirical studies of intron sequences [1], [9], [10], [17], [74] and simulation experiments [3], [16]. However, earlier studies examining the contribution of gap characters were confined to one or a few intron sequences and a single taxonomic range. By undertaking our comparisons across a range of evolutionary distances, involving alignments from the species- to the phylum level, we showed that gaps provide reliable source of phylogenetic information that is more conserved than base substitutions and, thus applicable across a wider range of phylogenetic questions above the level of interspecies comparisons.

Contrary to common belief, nuclear ribosomal internal transcribed spacers are useful for resolving phylogenetic relationships above species and genus level, when sophisticated alignment algorithms are employed and the indel data are involved in the phylogenetic reconstruction. Whether other analytical adjustments, e.g. partitioning the ITS locus into ITS1, 5.8S and ITS2 regions could further improve the precision of estimates remain to be tested. Nevertheless, we stress the need for accurate gap placement and indel coding in ITS alignments, in order to exploit as much of the phylogenetic information as possible. Due to the relentless progress in alignment estimation, the inference of biologically realistic indels is becoming increasingly plausible, which improves phylogenetic estimates drawn from rapidly evolving, indel-rich sequences. We believe that sophisticated alignment and indel handling strategies will paint a different picture on the phylogenetic utility of ITS and affect a number of analyses using ITS alignments; not only phylogeny reconstruction, but also other fields, such as species identification, environmental sequencing projects or barcoding and will facilitate a more insightful utilization of the marker that appears in more than two thirds of phylogenetic datasets, but has been perpetually criticized.

## Supporting Information

Figure S1 **Probability density of indel length distributions observed under various alignment methods.** Both parsimony informative and uninformative indels show a strongly left-skewed distribution, with the most abundant length category being single-residue indels. The right tail of the distributions (reaching its maximum at 2203 residues) have been truncated for better visualization.
(DOCX)

Figure S2 **Posterior probability ratios of congruent nodes.** Support values for relationships inferred both in analyses with and without the indel data are strongly biased towards those making use of indel characters (values>1).
(DOCX)

Table S1 **Fungal ITS datasets analyzed in this study.**
(DOC)

Table S2 **Predicted decay rate of the phylogenetic signal under different alignment and gap treatment methods, as a function of increasing evolutionary divergence.**
(DOCX)
