# Supplementary material for: Re-Mind the Gap! Insertion – Deletion Data Reveal Neglected Phylogenetic Potential of the Nuclear Ribosomal Internal Transcribed Spacer (ITS) of Fungi
Source: PLoS One. 2012 Nov 19;7(11):e49794. doi: 10.1371/journal.pone.0049794 (PMC3501463; doi:10.1371/journal.pone.0049794)

Fig. S1. Probability density of indel length distributions observed under various alignment methods. Both parsimony informative and uninformative indels show a strongly left-skewed distribution, with the most abundant length category being single-residue indels. The right tail of the distributions (reaching its maximum at 2203 residues) have been truncated for better visualization.


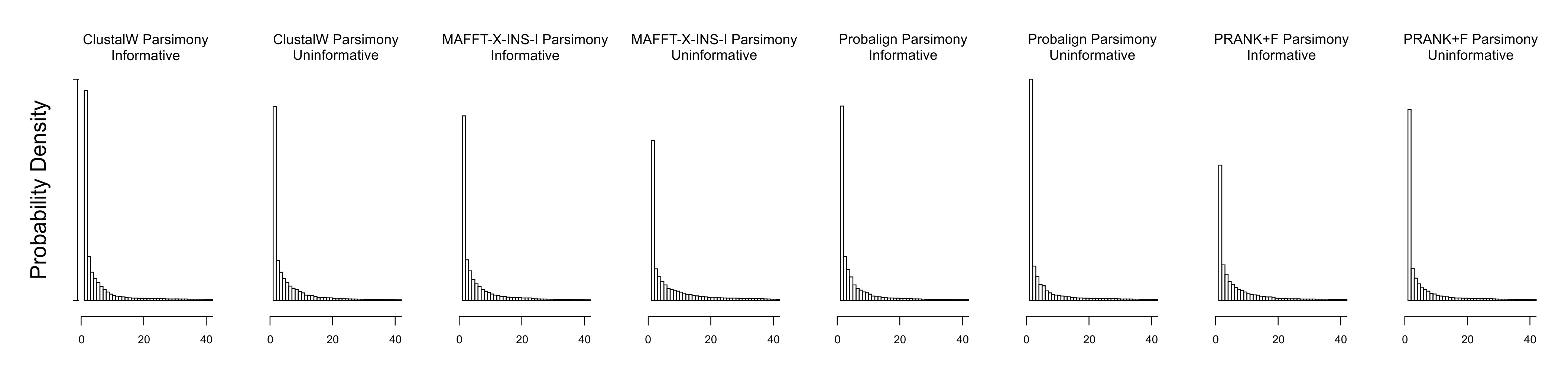

Supplement: Figure S1 — Probability density of indel length distributions observed under various alignment methods. Both parsimony informative and uninformative indels show a strongly left-skewed distribution, with the most abundant length category being single-residue indels. The right tail of the distributions (reaching its maximum at 2203 residues) have been truncated for better visualization. (DOCX) [file pone.0049794.s001.docx]
