# Supplementary material for: Re-Mind the Gap! Insertion – Deletion Data Reveal Neglected Phylogenetic Potential of the Nuclear Ribosomal Internal Transcribed Spacer (ITS) of Fungi
Source: PLoS One. 2012 Nov 19;7(11):e49794. doi: 10.1371/journal.pone.0049794 (PMC3501463; doi:10.1371/journal.pone.0049794)

Fig. S2. Posterior probability ratios of congruent nodes. Support values for relationships inferred both in analyses with and without the indel data are strongly biased towards those making use of indel characters (values > 1).


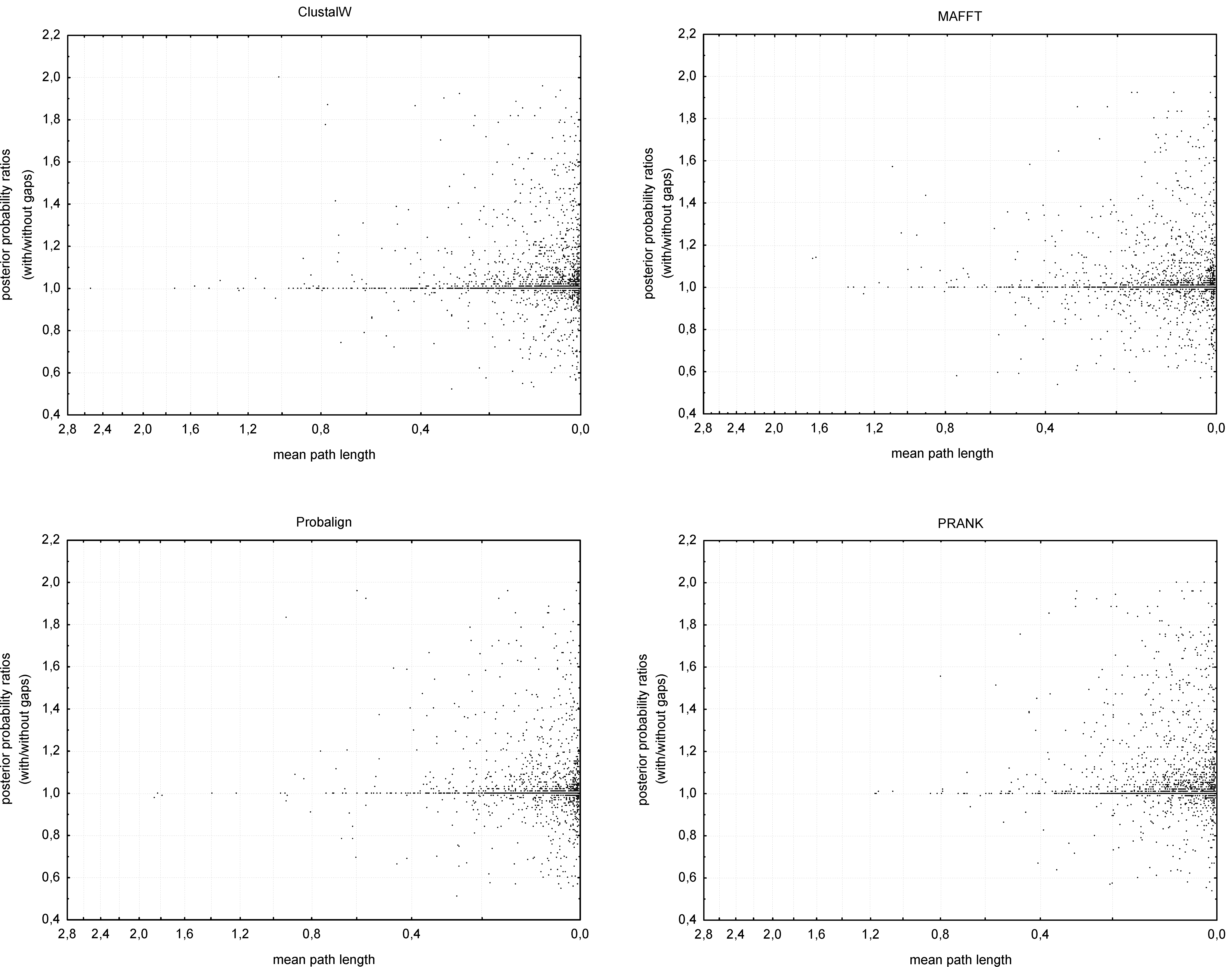

Supplement: Figure S2 — Posterior probability ratios of congruent nodes. Support values for relationships inferred both in analyses with and without the indel data are strongly biased towards those making use of indel characters (values>1). (DOCX) [file pone.0049794.s002.docx]
