# Supplementary material for: Re-Mind the Gap! Insertion – Deletion Data Reveal Neglected Phylogenetic Potential of the Nuclear Ribosomal Internal Transcribed Spacer (ITS) of Fungi
Source: PLoS One. 2012 Nov 19;7(11):e49794. doi: 10.1371/journal.pone.0049794 (PMC3501463; doi:10.1371/journal.pone.0049794)
Supplement: Table S1 — Fungal ITS datasets analyzed in this study. (DOC) [file pone.0049794.s003.doc]

**Supplementary Information**

Table S1. Fungal ITS datasets analyzed in this study.

| Dataset | Approximate taxonomic range[[1]](#footnote-2) | No. of taxa | No. of sequences | Classification | Reference |
| --- | --- | --- | --- | --- | --- |
| Abdollahzadeh_etal_2009 | family | 22 | 47 | Botryosphaeriales | Persoonia 23: 1–8. 2009 |
| Agaricaceae | family | 58 | 58 | Agaricales | M. Gube, Thesis |
| Albee-Scott_etal_2007 | order | 30 | 30 | Leucogastrales | Mycol. Res. 111: 653–662. 2007 |
| Amalfi_etal_2010 | genus | 23 | 47 | Hymenochaetales | Mycologia 102: 1303–1317. 2010 |
| Andjic_etal_2007 | genus | 15 | 29 | Mycosphaerellales | Mycol. Res. 111: 1184–1198. 2007 |
| Bakker_etal__2004 | genus | 22 | 33 | Boletales | Mycologia 96: 102–118. 2004 |
| Bates_etal_2009 | family | 49 | 49 | Agaricales | Fungal Diversity 37: 153-207. |
| Beugelsdijk_etal_2008 | species | 6 | 31 | Boletales | Persoonia 20: 1–7. 2008 |
| Bitzer_etal_2008 | family | 34 | 36 | Xylariales | Mycol. Res. 112: 251–270. 2008 |
| Boyle_etal_2006 | family | 56 | 107 | Agaricales | Mycol. Res. 110: 369 – 380. 2006 |
| Bruns_etal_2010 | Order | 48 | 134 | Boletales | Mycologia 102:438–446. 2010 |
| Bulman_etal_2009 | genus | 14 | 48 | Tuberales | Mycol Progress DOI 10.1007/s11557-009-0626-0. 2009 |
| Buyck_etal_2008 | family | 53 | 53 | Russulales | Fungal Diversity 28: 15-40. 2008 |
| Cai_etal_2006 | family | 50 | 50 | Sordariales | Mycol. Res. 110: 137 – 150. 2006 |
| Cai_etal_206 | family | 34 | 34 | Sordariales | Mycol. Res. 110: 359 – 368. 2006 |
| Choi_etal_2008 | species | 12 | 31 | Peronosporales | Mycol. Res. 112: 1327–1334. 2008 |
| Cooke_etal_2000 | genus | 50 | 50 | Peronosporales | Fungal Genetics and Biology 30: 17–32 2000 |
| Coprinus_patouillardii | species | 5 | 32 | Agaricales | This work (TreeBase: 12470) |
| Crewe_etal_2006 | genus | 18 | 33 | Acarosporales | Mycol. Res. 110: 521 – 52. 2006 |
| Crous_etal_2003 | order | 61 | 68 | Capnodiales | Sydowia 55: 136-152. |
| Danks_etal_2010 | genus | 156 | 174 | Agaricales | Persoonia 24: 106–126. 2010 |
| Diez_etal_2002 | genus | 7 | 17 | Pezizales | Mycologia, 94:247–259. 2002 |
| Druzhinina_etal_2005 | family | 79 | 79 | Hypocreales | Fungal Genetics and Biology 42: 813–828. 2005 |
| Eberhart_etal_2009 | genus | 9 | 31 | Agaricales | Mycol Progress DOI 10.1007/s11557-009-0627-z 2009 |
| Foos_etal_2010 | family | 9 | 19 | Mucorales | Mycologia 103:36. DOI: 10.3852/09-314. 2011 |
| Froeslev_etal_2005 | genus | 44 | 55 | Agaricales | Mol Phylogenet Evol. 37:602-18. 2005 |
| Gamper_etal_2009 | species/genus | ~13 | 57 | Diversisporales | New Phytologist 182: 495–506. 2009 |
| Garcia-Blásquez_etal_2008 | genus | 22 | 111 | Peronosporales | Mycol. Res. 112: 502–512. 2008 |
| Garnica_etal_2003 | genus | 55 | 87 | Agaricales | Mycologia 95: 1155-1170. 2003 |
| Ge_etal_2010 | species/genus | 12 | 51 | Agaricales | Fungal Diversity: 45:81–98. 2010 |
| Gonzáles_etal_2006 | species | 3 | 43 | Polyporales | Molecular Phylogenetics and Evolution 40 459–470. 2006 |
| Goodwin_etal_2001 | family | 28 | 29 | Capnodiales | Mycologia 93: 934-946. 2001 |
| Greslebin_etal_2004 | genus | 37 | 40 | Polyporales | Mycologia 96: 2004, pp. 260 –271 |
| Groenewald_etal_2011 | family | 37 | 40 | Saccharomycetales | Persoonia 26: 40–46. 2011 |
| Hirose_etal_2005 | genus | 9 | 47 | Erysiphales | Mycol. Res. 109: 912–922. 2005 |
| Hoffman_etal_2007 | genus | 18 | 42 | Mucorales | Mycol. Res. 111: 1169–1183. 2007 |
| Hofstetter_etal_2004 | family | 46 | 48 | Agaricales | Mycol. Res. 106: 1043–1059 2002 |
| Högnabba_etal_2006 | genus | 59 | 89 | Lecanorales | Mycol. Res. 110: 1080–1092. 2006. |
| HolstJensen_etal_1997 | family | 34 | 34 | Leotiales | Mycologia 89: 885-899 1997 |
| James_etal_2006[[2]](#footnote-3) | phylum | 49 | 49 | Basidiomycota | Nature 443, 818-822 2006 |
| Justo_etal_2010 | genus | 47 | 57 | Agaricales | Mycologia 102: 675–688. 2010 |
| Kemler_etal_2006 | family | 40 | 74 | Ustilaginales | BMC Evolutionary Biology 6:35. 2006 |
| Khodaparast_etal_2007 | genus | 18 | 37 | Erysiphales | Mycol. Res. 111: 673–679. 2007 |
| Kwasna_etal_2006 | order | 24 | 41 | Mucoromycotina | Mycol. Res. 110: 501–510. 2006 |
| Larsson&Örstadius_2008 | family | 56 | 68 | Agaricales | Mycol. Res. 112: 1165–1185. 2008 |
| Larsson_etal_2009 | genus | 40 | 81 | Agaricales | Persoonia 23: 86–98. 2009 |
| Larsson&Jeppson_2008 | genus | 49 | 78 | Agaricales | Mycol. Res. 112: 4–22. 2008 |
| Letcher_etal_2008 | order | 49 | 49 | Rhizophlyctidales | Mycol. Res. 112: 1031–1048. 2008 |
| Letcher_etal_2008 | order | 72 | 72 | Rhizophydiales | Mycol. Res. 112: 759–782. 2008 |
| Linnakoski_etal_2008 | species | 5 | 47 | Ophiostomatales | Mycol. Res. 112: 1475–1488. 2008 |
| Liou_etal_1997 | family | 27 | 34 | Orbiliales | Mycologia 89:876-884. 1997 |
| Luangsa-Ard_etal_2005 | genus | 29 | 43 | Hypocreales | Mycol. Res. 109: 581–589. 2005 |
| Lutzoni_etal_1997 | family | 30 | 30 | Agaricales | Syst. Biol. 46: 373-406. 1997 |
| Matheny_etal_2006/agaricales | Order | 54 | 54 | Agaricales | Mycologia 98:982–995. 2006 |
| Matheny_etal_2006/agaricoid clade | Order | 67 | 67 | Agaricales | Mycologia 98:982–995. 2006 |
| Matheny_etal_2006/hygrophoroid clade | Order | 24 | 24 | Agaricales | Mycologia 98:982–995. 2006 |
| Matheny_etal_2006/marasmioid clade | Order | 57 | 57 | Agaricales | Mycologia 98:982–995. 2006 |
| Matheny_etal_2006/pluteoid clade | Order | 13 | 13 | Agaricales | Mycologia 98:982–995. 2006 |
| Matheny_etal_2006/tricholomatoid clade | Order | 37 | 37 | Agaricales | Mycologia 98:982–995. 2006 |
| Mejia_etal_2008 | family | 29 | 29 | Diaporthales | Mycol. Res. 112: 23–35. 2008 |
| Miadlikovska_etal_2003 | genus | 33 | 82 | Peltigerales | Mycologia 95:1181–1203. 2003 |
| Miller_etal_2002 | genus | 89 | 89 | Russulales | Mycol. Res. 106: 259–276. 2002 |
| Moncalvo_etal_2008 | species | 9 | 60 | Polyporales | Mycol. Res. 112: 425–436. 2008 |
| Mortierella73 | family | 56 | 107 | Mortierellales | This work (TreeBase: 12470) |
| Muggia_etal_2008 | species | 33 | 43 | Teloschistales | Mycol. Res. 112: 36–49. 2008 |
| Naesborg_etal_2007 | family | 38 | 42 | Lecanorales | Mycol. Res. 111: 581–591. 2007 |
| Nagy et al 2012 | genus | 72 | 72 | Agaricales | Syst. Biol. in press. |
| Nagy_etal_2009 | genus | 13 | 40 | Agaricales | Persoonia 22: 28–37. 2009 |
| Nuytinct_etal_2007 | genus | 29 | 82 | Russulales | Mycol. Res. 111: 1285–1297. 2007 |
| Oda_etal_1999 | genus | 28 | 36 | Agaricales | Mycoscience 40: 57-64, 1999 |
| Oda_etal_2004 | species | 9 | 48 | Agaricales | Mycol. Res. 108: 885–896. 2004 |
| Olariaga_etal_2009 | species | 14 | 50 | Cantharellales | Mycol. Res. xxx 1–10. 2009 |
| Overton_etal_2006 | species | 16 | 37 | Hypocreales | STUDIES IN MYCOLOGY 56: 39–65. 2006 |
| Palice_etal_2005 | family | 30 | 31 | Hymenochaetales | Mycol. Res. 109: 447–451. 2005 |
| Peintner_etal_2001 | genus | 87 | 106 | Agaricales | American Journal of Botany 8812: 2168-2179. 2001 |
| Peintner_etal_2002 | genus | 39 | 45 | Agaricales | Mycologia 94: 620-629. 2002 |
| Peintner_etal_2003 | genus | 47 | 59 | Agaricales | Mycol. Res. 107: 485-494. 2002 |
| Petkovits_etal_2011 | order | 56 | 73 | Mortierellales | PLoS ONE 6(11): e27507. |
| Pildain_etal_2009 | genus | 10 | 74 | Agaricales | Mycol Progress 8:181–194. 2009 |
| Prieto_etal_2010 | genus | 11 | 28 | Verrucariales | Mycologia 102: 291–304. 2010 |
| Rainer_etal_2006 | species | 11 | 71 | Microascales | Mycol. Res. 110. 151 – 160. 2006 |
| Rungjindamai_etal_2008[[3]](#footnote-4) | family | 15 | 31 | Polyporales | Fungal Diversity 33: 139-161 |
| Rungjindamai_etal_2008[[4]](#footnote-5) | family | 24 | 43 | Polyporales | Fungal Diversity 33: 139-161 |
| Seena_etal_2010 | family | 21 | 105 | Helotiales | Fungal Diversity 44:77–87- 2010 |
| Seifert_etal_2007Fig6 | family | 48 | 55 | Chaetothyriales | PNAS 104: 3901–3906. 2007 |
| Selbman_etal_2008 | genus | 23 | 61 | Dothideales | Studies in Mycology 61: 1–20. 2008 |
| Seo_etal_2007 | order? | 49 | 52 | Ustilaginales | FEMS Yeast Res 7 1035–1045. 2007 |
| Solé_etal_2002 | family | 27 | 37 | Onygenales | Mycol. Res. 106: 388–396. 2002 |
| Stadler_etal_2010 | family | 46 | 46 | Xylariales | Persoonia 25: 11–21. 2010 |
| Stensrud_etal_2005 | family | 74 | 99 | Clavicipitales | Mycol. Res. 109: 41–56 2005 |
| Stockinger_etal_2009 | species | 4 | 143 | Glomales | New Phytologist 183: 1176–1187. 2009 |
| Stoll_etal_2003 | family | 49 | 56 | Ustilaginales | Canadian Journal of Botany, 81: 976-984. 2003 |
| Stoll_etal_2005 | family | 96 | 109 | Ustilaginales | Mycol. Res. 109: 342–356 2005 |
| Stubbe_etal_2010 | genus | 38 | 107 | Russulales | Fung. Biol. 114 271–283. 2010 |
| Suarez-Santiago_etal_2009 | genus | 10 | 41 | Agaricales | Mycol. Res. 113: 1070–1090. 2009 |
| Taylor_etal_2009 | family | 33 | 59 | Botryosphaeriales | Mycol. Res. 113: 337–353. 2009 |
| Telleria_etal_2010 | genus | 17 | 43 | Polyporales | Mycologia 102: 1426-1436. 2010 |
| Thell_etal_2004 | family | 93 | 93 | Lecanorales | Mycological Progress 3: 297–314. 2004 |
| Tomsovsky_etal_2010 | genus | 9 | 54 | Hymenochaetales | Mycol Progess 9: 225-233. 2010 |
| Trouillas_etal_2010 | genus | 16 | 57 | Diatrypales | Mycologia 102: 319–336. 2010 |
| Tsui_etal_2006 | family | 44 | 44 | Pleosporales | Mycologia 98: 94–104. 2006 |
| van Wyk_etal_2009 | genus | 24 | 76 | Ophiostomatales | Persoonia 22: 75–82. 2009 |
| Vellinga_etal_2003a | family | 55 | 55 | Agaricales | Mycologia 95: 442–456. 2003 |
| Vellinga_etal_2003lepi | genus | 79 | 87 | Agaricales | Mycological Progress 2: 305–322. 2003 |
| Vellinga_etal_2004 | family | 127 | 130 | Agaricales | Mycol. Res. 108 4 : 354–377. 2004 |
| Vellinga_etal_2007 | genus | 19 | 30 | Agaricales | Mycologia 99: 569–585. 2007 |
| Vellinga_etal_2009 | genus | 21 | 34 | Agaricales | Mycologia 102: 447–454. 2010 |
| Walker_etal_2010 | genus | 17 | 60 | Diaporthales | Mycologia 102:1479–1496. 2010 |
| Wannathes_etal_2008 | genus | 77 | 111 | Agaricales | Fungal Diversity 37: 209-306. 2008 |
| Wood_etal_2005 | family | 28 | 37 | Uredinales | Mycol. Res. 109: 387–400 2005 |
| Wu_etal_2000 | genus | 10 | 40 | Boletales | Molecular Phylogenet. Evol. 17:37–47. 2000 |
| Yang_etal_2005 | family | 47 | 76 | Agaricales | Mycol. Res. 109: 1259–1267 2005 |
| Zare_etal_2008 | genus | 36 | 81 | Hypocreales | Mycol. Res. 112: 811–824. 2008 |

1. the ranges are defined as (but see also Figure 3):

   Species level: with scope on relationships of a few species or intraspecific branching order.

   Genus level: including several species from the same genus

   Family level: includes multiple genera from of the family

   Order level: including representatives of several family

   Phylum: with a representative sample from the whole phylum [↑](#footnote-ref-2)
2. datasets contained only the Basidiomycetes clade [↑](#footnote-ref-3)
3. datasets corresponding to Fig. 4. [↑](#footnote-ref-4)
4. datasets corresponding to Fig. 5. [↑](#footnote-ref-5)
