# Supplementary material for: Re-Mind the Gap! Insertion – Deletion Data Reveal Neglected Phylogenetic Potential of the Nuclear Ribosomal Internal Transcribed Spacer (ITS) of Fungi
Source: PLoS One. 2012 Nov 19;7(11):e49794. doi: 10.1371/journal.pone.0049794 (PMC3501463; doi:10.1371/journal.pone.0049794)
Supplement: Table S2 — Predicted decay rate of the phylogenetic signal under different alignment and gap treatment methods, as a function of increasing evolutionary divergence. (DOCX) [file pone.0049794.s004.docx]

Table S2. Predicted decay rate of the phylogenetic signal under different alignment and gap treatment methods, as a function of increasing evolutionary divergence.

| Analysis | Regression Equation | R^2^ |
| --- | --- | --- |
| ClustalW, no gaps | - 0.02x+0.982 | 0.008 |
| ClustalW, with gaps | - 0.006x+0.914 | <0.0001 |
| MAFFT-X-INS-I, no gaps | - 0.04x+0.883 | 0.03 |
| MAFFT-X-INS-I, with gaps | - 0.044x+0.895 | 0.028 |
| Probalign, no gaps | - 0.091x+0.889 | 0.089 |
| Probalign, with gaps | - 0.026x+0.901 | 0.006 |
| PRANK_+F_, no gaps | - 0.021x+0.891 | 0.008 |
| PRANK_+F_, with gaps | 0.003x+0.937 | <0.0001 |
